# Supplementary material for: Evaluation of Food-Intake Behavior in a Healthy Population: Personalized vs. One-Size-Fits-All
Source: Nutrients. 2020 Sep 15;12(9):2819. doi: 10.3390/nu12092819 (PMC7551874; doi:10.3390/nu12092819)
Supplement: Supplementary file 1 [file nutrients-12-02819-s001.pdf]

Table S1. French National Nutrition and Health Program dietary guidelines shown per category.
A leaflet with these food categories was provided to the participants receiving GNA.

Food category	Recommendation
**Fruits and vegetables**	At least 5 portions per day (80g to 100g each), at each meal and in case of hunger
**Bread, cereals potatoes and legumes**	At each meal and according to appetite, promote whole grains and diversity
**Milk and dairy products**	3 units per day. Promote diversity and cheeses richest in calcium, with the lowest percentage fat and salt.
**Meat, poultry, fishery products and eggs**	1 to 2 units per day. In less quantity than the vegetables or cereals. For the meat: promote the variety of species and less fatty pieces. Consumption of fish at least twice a week.
**Added fat**	Limit the consumption. Promote variety and limit animal's origin fats (butter, cream)
**Sweet products**	Limit the consumption
**Beverages**	Water ad libitum. For the alcoholic beverages: in adults, do not exceed 2 glasses of wine per day (10 cl) for women and 3 for men. For example, 2 glasses of wine are equivalent to 2 halves of beer or 6 cl of strong alcohol
**Salt**	Limit the consumption. Do not add salt before tasting; reduce salt addition to cooking water.

Table S2. P-values of main and interaction effects for the 14 food categories in the participants receiving no nutritional advice (control), GNA, or PNA before (V2) and after the nine-week intervention period (V3).

Food Category	Time effect	Treatment effect	Treatment effect	Interaction effect	Interaction effect
	V2 vs. V3	PNA vs. control	PNA vs. GNA	PNA vs. control x Time	PNA vs. GNA x Time
**Fruits**	.	.	.	0.0004	0.01
**Vegetables**	0.20	0.29	0.29	0.76	0.16
**Whole grains**	.	.	.	0.02	0.07
**Pulses**	.	.	.	-	-
**Unsalted nuts**	.	.	.	<.0001	<.0001
**Dairy**	0.84	0.14	0.62	0.83	0.46
**Fish**	.	.	.	<.0001	<.0001
**Spreads and oils**	0.04	0.16	0.008	0.17	0.25
**Red meat**	0.41	0.64	0.91	0.49	0.91
**Cold cuts (meat)**	0.004	0.64	0.84	0.13	0.06
**SSB**	.	.	.	0.08	0.04
**Alcohol**	.	.	.	-	-
**Added salt**	.	.	.	0.004	0.007
**Unhealthy choices**	.	.	.	0.001	0.002

Control: n = 52, GNA: n = 52, PNA: n = 51. Note: the model could not fit the data of pulses and alcohol, therefore statistical analysis was not possible for these food categories. $P < 0.05$ was considered significant. GNA, generic nutrition advice; PNA, personalized nutrition advice; SSB, sugar sweetened beverages;

Table S3. P-values of the time and run in vs. intervention effects for the 14 food categories of the participants receiving PNA at baseline (V1), the end of the free living period (V2), and at the end of the study (V3).

Food Category	Time effect	Time effect	Run-in vs intervention effect
	PNA V1 vs V2	PNA V2 vs V3	PNA V2-PNA V1 vs PNA V3- PNA V2
**Fruits**	0.21	<.0001	0.06
**Vegetables**	0.85	0.34	0.41
**Whole grains**	0.36	0.008	0.009
**Pulses**	-	-	-
**Unsalted nuts**	0.36	<.0001	<.0001
**Dairy**	0.85	0.84	0.25
**Fish**	0.39	0.0003	0.25
**Spreads and oils**	0.84	0.09	0.42
**Red meat**	0.09	0.49	0.01
**Cold cuts (meat)**	0.30	0.01	0.006
**SSB**	0.52	0.005	0.01
**Alcohol**	-	-	-
**Added salt**	0.49	0.003	0.009
**Unhealthy choices**	0.34	0.002	0.46

PNA: $n = 51$. This Table refers to Supplemental Figure 3. Note: the model could not fit the data of pulses and alcohol, therefore statistical analysis was not possible for these food categories. $P < 0.05$ was considered significant. PNA, personalized nutrition advice; SSB, sugar sweetened beverages.

Table S4. P-values of main and interaction effects for the 10 food categories where participants in the PNA-group set goals for before (V2) and after the nine week intervention period (V3).

	Participants who didn't set a goal	Participants who set a goal	Time effect	Goal effect	Interaction effect
**Food category**	n	n			Goal vs no goal
**Unsalted nuts**	21	29	.	.	<.0001
**Fruits**	27	23	.	.	0.004
**Whole grains**	32	18	.	.	0.001
**Fish**	34	16	.	.	<.0001
**Spreads and oils**	39	11	0.15	0.95	0.42
**Dairy**	41	9	.	.	<.0001
**Added Salt**	42	8	-	-	-
**Vegetables**	42	8	.	.	0.0007
**Cold cuts (meat)**	44	6	-	-	-
**Unhealthy food choices**	44	6	.	.	0.03

PNA: $n = 50$ who set a goal. $P < 0.05$ was considered significant. PNA, personalized nutrition advice.
